# Role of meteorological conditions in reported chickenpox cases in Wuhan and Hong Kong, China

**DOI:** 10.1186/s12879-017-2640-1

**Published:** 2017-08-03

**Authors:** Banghua Chen, Ayako Sumi, Lei Wang, Wang Zhou, Nobumichi Kobayashi

**Affiliations:** 1 Department of Infectious Diseases Control and Prevention, Wuhan Center for Disease Control and Prevention, Wuhan, Hubei China; 2 Department of Hygiene, Sapporo Medical University School of Medicine, S-1, W-17, Chuo-ku, Sapporo, Hokkaido 060-8556 Japan; 3 Institute of Infectious Disease Control and Prevention, Hubei Center for Disease Control and Prevention, Wuhan, China; 4 Wuhan Centers for Disease Control and Prevention, 24 Jianghanbei Road, Wuhan, Hubei 430000 China

**Keywords:** Chickenpox, Seasonality, Temperature, Rainfall, Time-series analysis

## Abstract

**Background:**

Chickenpox is a common contagious disease that remains an important public health issue worldwide. Over 90% of unvaccinated individuals become infected, but infection occurs at different ages in different parts of the world. Many people have been infected by 20 to 30 years of age in China, and adults and pregnant women who become infected often develop severe infection. Furthermore, a mortality rate of 2–3 per 100,000 infected persons has been reported. In this study, we explore the temperature-dependent transition of patterns of reported chickenpox cases in two large subtropical climate cities, Wuhan and Hong Kong, China, to aid in the prediction of epidemics and preparation for the effects of climatic changes on epidemiology of chickenpox in China.

**Methods:**

We used a time series analysis comprising a spectral analysis based on the maximum entropy method in the frequency domain and the nonlinear least squares method in the time domain. Specifically, the following time series data were analyzed: data of reported chickenpox cases and meteorological data, including the mean temperature, relative humidity and total rainfall in Wuhan and Hong Kong from January 2008 to June 2015.

**Results:**

The time series data of chickenpox for both Wuhan and Hong Kong have two peaks per year, one in winter and another in spring, indicating a bimodal cycle. To investigate the source of the bimodal cycle of the chickenpox data, we defined the contribution ratio of the 1-year cycle, *Q*_1_, and the 6-month cycle, *Q*_2_, as the contribution of the amplitude of a 1-year cycle and a 6-month cycle, respectively, to the entire amplitude of the time-series data. The *Q*_1_ values of Wuhan and Hong Kong were positively correlated with the annual mean temperature and rainfall of each city. Conversely, the *Q*_2_ values of Wuhan and Hong Kong were negatively correlated with the annual mean temperature and rainfall of Wuhan and Hong Kong.

**Conclusion:**

Our results showed that the mean temperature and rainfall have a significant influence on the incidence of chickenpox, and might be important predictors of chickenpox incidence in Wuhan and Hong Kong.

**Electronic supplementary material:**

The online version of this article (doi:10.1186/s12879-017-2640-1) contains supplementary material, which is available to authorized users.

## Background

Chickenpox (varicella), which is caused by the ubiquitous varicella-zoster virus, is an extremely contagious vaccine-preventable infectious disease [1–4]. Over 90% of unvaccinated individuals become infected, but infection occurs at different ages in different parts of the world. For example, over 80% of people have been infected by 10 years of age in the United States, the United Kingdom, and Japan, while many people have been infected by 20 to 30 years of age in China, India, Southeast Asia, and the West Indies [5]. In these areas, adults and pregnant women who become infected often develop severe infection. Furthermore, a mortality rate of 2–3 per 100,000 infected persons has been reported [6]. Therefore, chickenpox remains an important public health issue, especially in China, India, Southeast Asia, and the West Indies.

Potential transmission of infectious diseases including chickenpox is believed to be affected by changes in climate, and some studies have examined the relationship between weather variability and the incidence of infectious diseases [4, 7, 8]. We previously demonstrated that temperature was associated with reported chickenpox incidence in Japan [3]. Therein, bimodal cycles of reported chickenpox incidences that were clearly observed in northern Japan disappeared at lower latitudes, and unimodal cycles appeared in southern Japan. This transition of patterns of reported chickenpox incidences in Japan was considered to be temperature-dependent.

The present study was conducted to explore whether the temperature-dependent transition of patterns of reported chickenpox incidences in Japan is applicable to areas in which chickenpox incidences are a serious problem, such as China, India, Southeast Asia, and the West Indies. In the present study, we investigated the seasonality of reported chickenpox cases in Hong Kong and Wuhan, China, where a nationwide internet-based infectious diseases reporting system has been established and accumulated good-quality surveillance data on chickenpox [9]. The analyses included spectral analyses conducted using the maximum entropy method (MEM) and the least squares method (LSM) [3, 8]. The results presented herein will facilitate prediction of epidemics and preparation for the effects of climatic changes on the epidemiology of chickenpox in China.

## Methods

### Data

#### Chickenpox data

Chickenpox is considered an easily diagnosed childhood disease due to the presence of an often pathognomonic rash; therefore, it is rarely subject to misdiagnosis and miscoding [10, 11]. Case reports of chickenpox in Wuhan and Hong Kong were defined by medical doctors as acute generalized maculopapular vesicular rashes without other apparent cause [4, 12].

#### Wuhan

Wuhan has an area of 8494 km^2^ and a population of 10 million (as of 2015) [13]. As of 2015, the age distribution of the Wuhan population was as follows: infants and preschool children, aged ≤4 years (3.6%); pre-teens, aged 5–9 years (3.8%); teens, aged 10–19 years (10.2%); and adults, aged ≥20 years (82.4%). Though chickenpox was not one of the notifiable infectious diseases in China, it was categorized as a surveillance infectious disease by the Wuhan Department of Health in 2006, after which hospitals in Wuhan were required to report chickenpox cases on-line to the China Information System for Disease Control and Prevention using a standardized form. In this study, we analyzed the weekly number of cases of chickenpox reported in Wuhan between January 2008 and June 2015 (390 data points). The data are available from the CISDCP website through the Wuhan Centers for Disease Control and Prevention.

#### Hong Kong

Hong Kong has an area of 1104 km^2^ [13] and a population of 7.3 million (as of 2015) [14]. As of 2015, the age distribution of the Hong Kong population was as follows: infants and preschool children, aged ≤4 years (3.8%); pre-teens aged 5–9 years (4.0%); teens, aged 10–19 years (8.3%); and adults, aged ≥20 years (83.9%) [14]. In Hong Kong, China, chickenpox is a notifiable infectious disease; therefore, all medical practitioners in both public and private sectors are required to report all cases to the Centre of Health Protection (CHP), Department of Health, Hong Kong Special Administrative Region Government via a centralized notification system. The monthly number of chickenpox cases were obtained from the CHP for January 2008 to June 2015 (90data points) (http://www.chp.gov.hk).

### Meteorological data

Using daily temperature, relative humidity, rainfall, and wind velocity data for 2008–15 for Wuhan and for Hong Kong [15], we calculated mean values, which corresponded to average daily data (one data point). We also calculated the total daily rainfall for the two places for 2008–15 (one data point).

#### Wuhan

Wuhan is located in a subtropical wet monsoon climate area in which there is heavy rainfall and four clearly defined seasons. Seasons were defined as spring (April), summer (May–September), autumn (October) and winter (November–March). Daily meteorological data, including the mean temperature, relative humidity and total rainfall, were collected in the study region by the Meteorological Department, Wuhan, which received and managed real-time data from 116 meteorological surveillance sites widely distributed in Wuhan. The daily data were gathered for a total of 2922 days from January 2008 to June 2015 (2922 data points).

#### Hong Kong

Hong Kong is located in eastern Asia, bordering the South China Sea to the south, west, and east, and mainland China to the north. It has a subtropical and monsoon-influenced climate with wet and hot summers and dry and cool to mild winters [8]. Seasons are defined as spring (March–May), summer (June–August), autumn (September–November), and winter (December–February). Meteorological data including daily mean temperature, mean relative humidity and rainfall were obtained from January 2008 to June 2015 from daily meteorological reports from the Hong Kong Observatory website [15]. We obtained daily data for a total of 2922 days from January 2008 to June 2015 (2922 data points).

### Vaccine

Chickenpox vaccine became available in Wuhan in 2006. Parents wishing to have their children vaccinated must pay for the service. The estimated chickenpox vaccination rate was less than 10% in children aged under 6 years, according to vaccine information from Wuhan Central Disease Control.

In Hong Kong, chickenpox vaccines have been incorporated in the region’s universal Childhood Immunisation Programme as a free, optional vaccine since July 2014 for children born on or after January 1, 2013. Children are supposed to receive two doses of the vaccine. The first dose is given at age 1 year. The second dose is given when the children enter the first year of primary school [16]. Before the implementation of universal varicella vaccination, children in Hong Kong were able to receive chickenpox vaccination only through the private sector. A survey conducted by Hong Kong’s Department of Health reported that only 32.4% of locally born children had received varicella vaccine in 2009 [17]. A 2012 survey of students aged 2–6 years in Hong Kong kindergartens revealed that the varicella vaccination rate was 57.6% [18]. Although varicella vaccine coverage has increased in Hong Kong, it remains below that of the United States (79.9%–92.0%) [19].

### Data analysis

The effects of meteorological conditions (temperature, relative humidity, rainfall) on the reported number of chickenpox cases were estimated as previously described [3]. An outline of the analysis procedure is as follows.

1. *Setting up time-series data for the analysis*: equal sampling time intervals of the original time series data *x*(*t*) (where *t* = time) are chosen, lack of *x*(*t*) compensated for, outliers corrected, logarithm transformation performed, and removal of long-term trend of *x*(*t*) performed, if necessary.
2. *MEM spectral analysis*: spectral analysis in the frequency domain is conducted based on the maximum entropy method (MEM), and the power spectral density (PSD) is obtained. From the result of PSD, periodic modes constructing seasonality of *x*(*t*) are assigned. The formulation of MEM-PSD is described in the reference [8].
3. *Least squares method (LSM)*: the validity of the MEM spectral analysis results was confirmed by calculation of the least squares fitting (LSF) curve to *x*(*t*) with MEM estimated periods. The formulation of the LSF curve in *X* (*t*) is described as follows:
  1 $$ X(t)={A}_0+\sum_{n=1}^N{A}_n \cos \left\{2\pi {f}_n\left(t+{\theta}_n\right)\right\}, $$

which is calculated using the LSM for *x*(*t*) with unknown parameters *f*_*n*_, *A*_0_ and *A*_n_ (*n* = 1, 2, 3, …, *N*), where *f*_*n*_ (=1/*T*_*n*_; *T*_*n*_ is the period) is the frequency of the *n*-th component, *A*_0_ is a constant that indicates the average value of the time series data, *A*_*n*_ and *θ*_*n*_ are the amplitude and the phase of the *n*-th component, respectively, and *N* is the total number of components.

(4) *Contribution ratio*: a ‘contribution ratio’ was defined for assignment of periodic modes constructing the seasonality of the original time series data *x*(*t*) [20–24]. The contribution ratio *Q*_*n*_ is described as follows:
  2 $$ {Q}_n=\raisebox{1ex}{${A}_n^2$}\!\left/ \!\raisebox{-1ex}{$Q$}\right., $$

where, *A*_*n*_ indicates the amplitude of the *n*-th periodic mode constituting the LSF curve *X*(*t*) to the original data *x*(*t*) [Eq. (1)], and *Q* is the total power of *x*(*t*).

## Results

### Age distribution

**Fig. 1:**
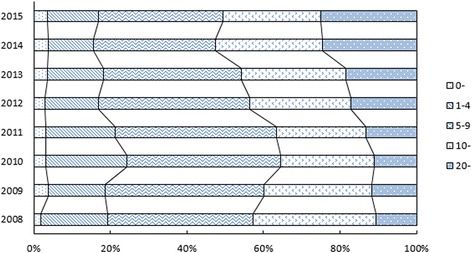
Age distribution of reported chickenpox cases in Wuhan

From January 2008 to June 2015, 32,671 cases and 72,295 cases of chickenpox were reported in Wuhan and Hong Kong, respectively. The age distribution of the reported cases in Wuhan is shown in Fig. 1. In Wuhan, chickenpox cases mainly affected schoolchildren and adolescents (5–19 years). During the study period, 32%–42% and 23%–32%, respectively, of all cases were reported in individuals aged 5–9 and 10–19 years. Also in Wuhan, 18,815 (58%) male and 13,856 (42%) female cases of chickenpox were reported. We did not have access to age and sex distribution data concerning reported cases of chickenpox in Hong Kong.

### Temporal variations in reported chickenpox incidence

**Fig. 2:**
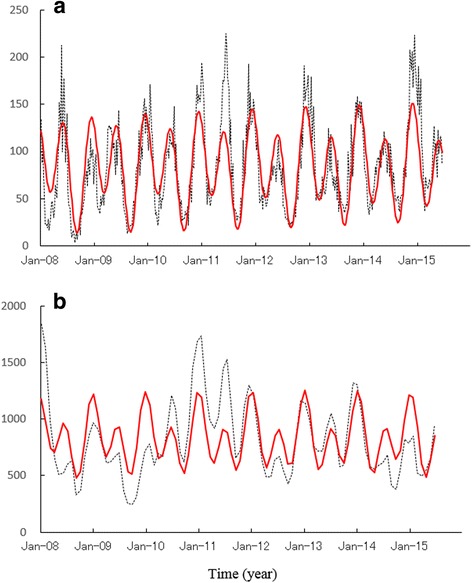
Comparison of least-squares fitting curves calculated for 1.0- and 0.5-year periodic modes (*red solid line*) with original data (*black dotted line*) from January 2008 to June 2015 for: **a** Wuhan (weekly data), **b** Hong Kong (monthly data)

Figure 2 indicates the weekly reported chickenpox incidence data for Wuhan (*a*) and Hong Kong (*b*). A 1-year chickenpox data cycle is shown for each location. For Wuhan (Fig. 2*a*), two peaks, one in winter and another in spring, were superimposed on a 1-year cycle. This bi-modal cycle was clearly observed in the case of Hong Kong (Fig. 2*b*).

### Spectral analysis and LSF analysis

**Fig. 3:**
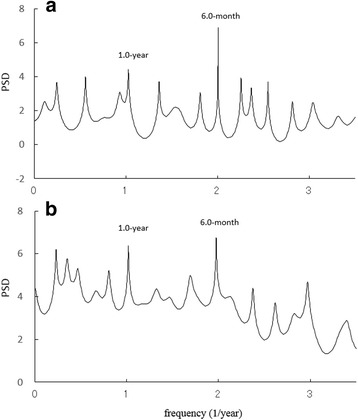
Power spectral density of the original data for: **a** Wuhan, **b** Hong Kong

The PSDs based on the MEM for chickenpox data (Fig. 2) are shown in Fig. 3. In each PSD, the prominent spectral line was observed at *f* = 1.0 [*f*(1/year); frequency] (=*f*_1_) corresponding to the 1-year cycle, and the spectral lines of *f*_2_ (=*f*_1_ × 2) corresponding to the 6 month cycle were observed at *f* = 2.0. To investigate the seasonality of each set of chickenpox data (Fig. 2) in detail, the LSF curve in Eq. (1) was calculated for the 1-year and 6-month cycles that were clearly observed in the PSD (Fig. 3). The LSF curves are shown in Fig. 2. In the figure, both LSF curves demonstrated a bi-modal seasonal cycle with the first peak in winter (December–January) and the second peak in spring (May–July). The contribution ratios [Eq. (2)] of the 1-year cycle (Q_1_) for Wuhan and Hong Kong were 0.13 and 0.21, respectively. The contribution ratios of the 6-month cycle (Q_2_) for Wuhan and Hong Kong were 0.65 and 0.39, respectively. The summation of the *Q*_1_ and *Q*_2_ values for Wuhan and Hong Kong were 0.78 (corresponding to 78%) and 0.60 (corresponding to 60%), respectively, indicating that these cycles made major contributions to the seasonal oscillations.

### Correlation between reported chickenpox incidences and meteorological conditions

**Fig. 4:**
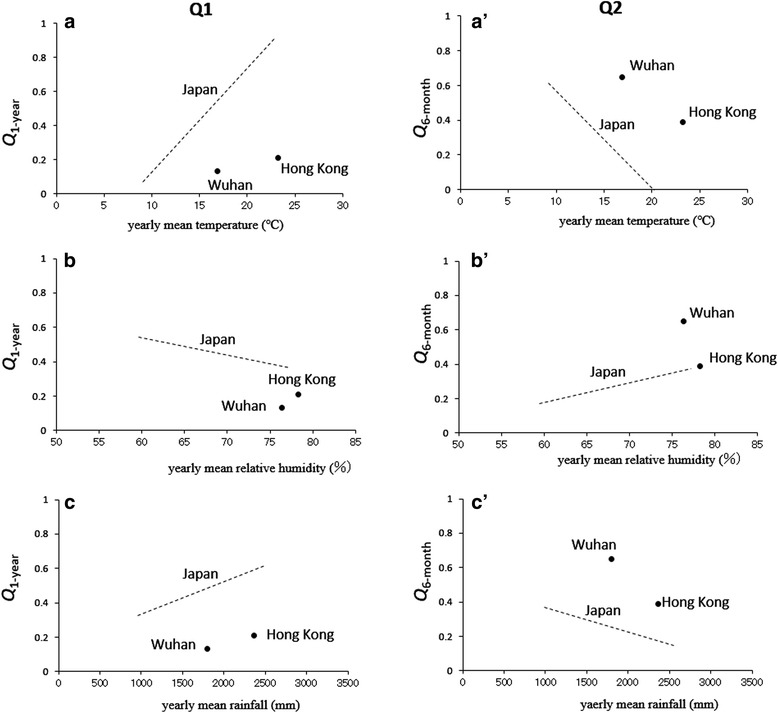
A plot of *Q*_1_ (*left-hand side*) and *Q*_2_ (*right-hand side*) against meteorological factors in Wuhan and Hong Kong from January 2008 to June 2015. *Dashed lines* indicating results from 47 prefectures in Japan [Fig. 6 in ref. 3] were shown for comparison to the present results of Wuhan and Hong Kong. **a** and (**a’**) weekly and monthly mean temperature (°C) for Wuhan and Hong Kong, respectively; (**b**) and (**b’**) weekly and monthly mean relative humidity (%) for Wuhan and Hong Kong, respectively; (**c**) and (**c’**) summation of weekly and monthly rainfall (mm) for Wuhan and Hong Kong, respectively

Figure 4*a*–*c* and Fig. 4*a’*–*c’* show plots of the contribution ratio of the 1-year cycle (*Q*_1_) and the 6-month cycle (*Q*_2_), respectively, to the mean temperature and relative humidity and a summation of the rainfall data for January 2008–June 2015. These figures also show results reported for Japan in a previous study [3]. The meteorological data for Wuhan and Hong Kong presented in Fig. 4 are listed in Table 1*a*, *b*, *d*.

**Table 1:** The value of mean, standard deviation (SD) and SD/mean for daily temperature (a), daily relative humidity (b), daily rainfall (c), and (d) the value of summation of the daily rainfall during 2008–2015 for Wuhan and Hong Kong

	a. Daily temperature			b. Daily relative humidity			c. Daily rainfall			d. Summation of the daily rainfall
	mean (°C)	SD (°C)	SD/mean	mean (%)	SD (%)	SD/mean	mean (mm)	SD (mm)	SD/mean	
Wuhan	18.9	9.2	0.49	76.4	8.6	0.11	34.9	116.2	3.33	1797.8
Hong Kong	23.2	4.9	0.21	78.3	6.3	0.08	6.4	20.0	3.12	2363.8

#### Temperature

As shown in Fig. 4*a*, the *Q*_1_ values of Wuhan and Hong Kong were positively correlated with the mean annual temperatures of Wuhan (16.9 °C) and Hong Kong (23.2 °C). This trend was essentially the same as for Japan (Fig. 4 *a*), where the value of *Q*_1_ increased as the annual mean temperature increased. The *Q*_1_ values for Wuhan and Hong Kong were smaller than those for prefectures in Japan in the same yearly mean temperature range.

As shown in Fig. 4*a’*, the *Q*_2_ values of Wuhan and Hong Kong were negatively correlated with the annual mean temperature of Wuhan (16.9 °C) and Hong Kong (23.2 °C). This trend is essentially same as that observed in Japan (Fig. 4 *a’*), where the *Q*_2_ value decreased as the annual mean temperature increased. The *Q*_2_ values for Wuhan and Hong Kong were larger than those for prefectures in Japan in the same yearly mean temperature range.

#### Relative humidity

For Wuhan and Hong Kong, the *Q*_1_ (Fig. 4*b*) and *Q*_2_ (Fig. 4*b’*) values were correlated with the annual mean relative humidity when it ranged from 76.4% to 78.3%, while the *Q*_1_ and *Q*_2_ values for Japan were correlated with relative humidity of 59.3% to 77.2% (Figs. 4*b* and b’). No clear trends in variations of *Q*_1_ and *Q*_2_ were observed for Wuhan and Hong Kong.

#### Rainfall

For the annual mean rainfall (Fig. 4*c*), the *Q*_1_ values of Wuhan and Hong Kong were positively correlated with the annual mean rainfall of Wuhan (1798 mm) and Hong Kong (2364 mm). This trend was essentially the same as observed for Japan (Fig. 4*c*), where the value of *Q*_1_ increased as the annual mean rainfall increased. The values of *Q*_1_ for Wuhan and Hong Kong were smaller than those for prefectures in Japan in the same yearly mean rainfall range.

For the annual mean rainfall (Fig. 4*c*’), the values of *Q*_2_ of Wuhan and Hong Kong were negatively correlated with the annual mean rainfall of Wuhan (1798 mm) and Hong Kong (2364 mm). This trend is essentially the same as that observed for Japan (Fig. 4*c*), where the *Q*_2_ value decreased as the annual mean rainfall increased. The *Q*_2_ values for Wuhan and Hong Kong were larger than those for prefectures in Japan in the same yearly mean rainfall range.

## Discussion

The results of the present study revealed that the *Q*_1_ and *Q*_2_ of temperatures for Wuhan and Hong Kong (Figs. 4a and a’) were essentially same as for Japan. In our preceding study, bimodal cycles of reported chickenpox incidences that were clearly observed in areas of northern Japan such as Hokkaido (latitude 43°N) disappeared at lower latitudes, and unimodal cycles appeared in Okinawa Prefecture, the most southern prefecture in Japan (latitude 26°N). This transition of patterns of reported chickenpox incidences in Japan was considered to be temperature-dependent. Thus, it is reasonable that temporal patterns of chickenpox incidences observed for Wuhan and Hong Kong (Fig. 2) were dominated by temperature as well.

The trends of *Q*_1_ and *Q*_2_ of temperature for Wuhan and Hong Kong (Fig. 4a and a’) support the findings reported by Shoji et al. [25], who showed that the reported cases of chickenpox increased at 5 °C–20 °C (i.e., the temperature range at which the chickenpox virus is activated) and decreased at temperatures lower than 5 °C and higher than 20 °C. In Wuhan, where the temperature falls below 5 °C in winter and exceeds 20 °C in summer, the occurrence of epidemics is bimodal (Fig. 2a). In contrast, the occurrence of epidemics is expected to be unimodal in Hong Kong, where the temperature rarely falls below 5 °C in winter; however, it was actually bimodal (Fig. 2b). This bimodal cycle of chickenpox epidemics in Hong Kong may be related to the fact that the values of *Q*_2_ for Hong Kong are larger than those for Japanese prefectures with the same mean temperature (Fig. 4a’). One study revealed a spring peak in a bimodal pattern of chickenpox cases related to spring vacation [26]. With respect to Wuhan and Hong Kong, school children do not have spring vacation; thus, it is unlikely that the occurrences of bimodal reported chickenpox incidences in Wuhan and Hong Kong are related to the school calendar. Rather, the bimodal pattern likely results from the mean temperature, as shown in Fig. 4a’.

The increase in the magnitude of *Q*_2_ for Wuhan and Hong Kong (Fig. 4a’) may be explained in terms of (i) the age distribution of reported chickenpox cases, as well as (ii) the effect of rainfall.

*(i) Age distribution of reported chickenpox cases*: For Hong Kong, more than 90% of the notifications were regarding children aged <18 years, with 29.1% of notified pediatric cases of chickenpox receiving treatment at public or private hospitals [27]. For Wuhan and Hong Kong, the proportion of reported cases among school children was high relative to Japan. Specifically, the 5–19 year age group accounted for 60%–70% of all cases from 2007 to 2015 for Wuhan and the 6–17 year age group accounted for 52.4% of all cases for Hong Kong [18], while pre-school children (0–4 years) accounted for 78% of the cases reported during 2009–2011 for Japan [3]. For Wuhan and Hong Kong, it can be assumed that school children tend to stay indoors during the day, which in turn increased domestic transmission. Given the limited indoor space in schools in Wuhan and Hong Kong, school children may have a greater opportunity for close contact, facilitating chickenpox transmission. In addition, the high population densities of Wuhan (1248 persons/km^2^) and Hong Kong (6622 persons/km^2^) may make it easier for chickenpox to spread from one person to another.

*(ii) Effect of rainfall*: It has been reported that rainfall was positively associated with chickenpox notifications in Hong Kong [2]. Indeed, the annual mean summation of rainfall in Hong Kong (2364 mm) is larger than that of Wuhan (1798 mm) and 43 of the 47 prefectures in Japan (Fig. 4c’). During the hot and wet season with high temperature and heavy rain, children tend to stay indoors with air-conditioning, which in turn increases domestic transmission. Given the limited indoor space in Hong Kong and Wuhan, children may have a greater opportunity for close contacts with one another, facilitating chickenpox transmission. However, the exact mechanism for the association between rainfall and chickenpox notifications remains unclear.

Critselis et al. examined the influence of meteorological conditions on chickenpox in Greece; before introduction of the vaccine, the authors found that the occurrence of hospitalized chickenpox cases was positively associated with wind speeds of 2.7–3.5 m/s [28]. We confirmed that for Wuhan, the *Q*_1_ and *Q*_2_ values were, respectively, 0.13 and 0.63 with an annual mean wind speed of 1.8 m/s; for Japan’s 47 prefectures, the mean wind speed appeared to display a randomly scattered pattern (Additional file 1).

As with Japan, the *Q*_1_ and *Q*_2_ values for Wuhan and Hong Kong are evidently dominated by temperature [3]; the meteorological data appear in Table 1. In Table 1c, there is clearly large variance in the daily rainfall data for Wuhan and Hong Kong. The amount of rainfall depends on the amount of water vapor in the atmosphere, which influences relative humidity [29]. The variation in the relative humidity for Wuhan and Hong Kong (Table 1b) was relatively small compared with that for rainfall (Table 1 *c*). This finding is the result of relative humidity being constrained by the amount of saturated water vapor, which is dependent on air temperature [29]. Thus, it is reasonable to infer that the unimodal and bimodal cycles observed in temporal variations of the reported chickenpox incidence were dominated by temperature.

There was a large difference between vaccine coverage in Wuhan (10%) and Hong Kong (57.6%); however, the temporal patterns of the incidence data for the two areas indicated approximately the same pattern, i.e., the bimodal cycle (Fig. 2 *a*, *b*). Thus, the vaccination coverage rate may not have affected the temporal patterns of the incidence data during the period of the present study (2008–15). In a previous investigation, we confirmed that the bimodal cycle of chickenpox epidemics observed in Japan was independent of the influence of the vaccination coverage rate (20%–40%) [3]. If the vaccination coverage rate exceeded 80% in Wuhan, Hong Kong, and Japan, the seasonal peak superposed on a 1-year cycle would diminish; that phenomenon has been observed in the United States [19].

It is possible that the epidemiology of chickenpox in subtropical Wuhan and Hong Kong differs from that in temperate Japan. In a previous study, we found that preschool children (aged ≤4 years) in Japan accounted for over 70% of all chickenpox cases in 2000–11 [3]. In Wuhan, chickenpox typically occurs at a later age, with many cases among schoolchildren and adolescents (5–19 years). The relatively low number of reported chickenpox cases among preschool children (≤4 years) in Wuhan may be the result of the reduced chickenpox virus transmission reported for tropical areas [30]. Garnett et al. [31] proposed that in tropical regions, the transmission potential of the chickenpox virus could be adversely affected by a combination of high ambient temperatures and humidity. For example, outbreaks of chickenpox in India appear to be more common in the cooler than in the warmer months of the year [32]. By contrast, in equatorial regions, such as Singapore, the incidence does not seem to vary according to the time of year [33].

Recently, Rice [34] interpreted the seasonality of the reported incidence of chickenpox in tropical regions with respect to levels of ultraviolet radiation and air pollution. Accordingly, the effect of meteorological factors on chickenpox incidence may differ from one country to another in different climate regions. To determine the underlying causes of chickenpox virus transmission in each climate region and effectively utilize the obtained results for health services, it is necessary to conduct a systematic study; the investigation should quantify the impact of meteorological factors on the chickenpox incidence in various countries in each climate region. Toward that end, the susceptible-exposed-infected-recovered (SEIR) model, which is a well-known mathematical model of infectious disease epidemics, may be appropriate; it has been shown to be effective with measles [35]. Thus, we would expect that a theoretical procedure, such as the SEIR model, could contribute to future investigations into the meteorological factors related to chickenpox transmission [35].

It is important to assess the sensitivity and representativeness of incidence data, as was performed by Souty et al. [36] for chickenpox in France. However, the CISDCP in Wuhan and CHP in Hong Kong have not announced the relevant information (such as age, location, and number of reported severe cases of chickenpox) that would allow us to determine the sensitivity and representativeness of the data used in the present study. There should be increased awareness of the information needed to determine the sensitivity and representativeness of the data in the surveillance system; that would allow a more accurate estimate of the burden of chickenpox and help prevent it in Wuhan and Hong Kong.

It should be noted that this study was limited in that, for Hong Kong, we used monthly data for chickenpox, while we used weekly data for Wuhan, because monthly measures are the minimum unit of measurement released by the Hong Kong Observatory website. We investigated the Q_1_ and Q_2_ values for the monthly data for Wuhan, by converting the weekly data for Wuhan into monthly data to enable comparisons with the results shown in Fig. 4. Using the monthly data, we confirmed that the Q1 and Q2 values (0.14 and 0.68, respectively) were essentially consistent with the weekly data (0.13 and 0.65, respectively). Thus, we concluded that the effect on the results of using monthly data compared with weekly data for Wuhan was negligible (Additional file 2). Further studies using weekly data for Hong Kong should be conducted to address the issue of the relationship between climate patterns and chickenpox epidemiology.

## Conclusion

We confirmed that mean temperature and rainfall have a significant influence on the incidence of chickenpox, and that these factors might be important predictors of chickenpox incidence in Wuhan and Hong Kong. Further time-series analyses of chickenpox and meteorological data from other regions in China may help determine the disease’s potential relationship to epidemiological factors.

## Additional files

Additional file 1: Plot of *Q*_1_ and *Q*_2_ against mean wind velocity. The black filled circles show *Q*_1_ for Japan’s 47 prefectures, the black open circles *Q*_2_ for Japan’s 47 prefectures, the red filled circles *Q*_1_ for Wuhan, and red open circles *Q*_2_ for Wuhan. (DOCX 23 kb)

Additional file 2: Plot for *Q*_1_ and *Q*_2_ of the monthly data for Wuhan compared with the results obtained with the corresponding weekly data shown in Fig. 4. The red filled circles indicate *Q*_1_ and *Q*_2_ for Wuhan’s monthly data. To convert the original weekly data into monthly data, the weekly data extending across two months from the end of the first month to the beginning of the next month were decomposed into daily data by calculating the mean daily values of the weekly data. Then, we obtained monthly data by adding the daily data thus obtained and the remaining weekly data. (DOCX 46 kb)

## Abbreviations

CISDCP: China information system for disease control and prevention
LSF: Least squares fitting
LSM: Least squares method
MEM: Maximum entropy method
PSD: Power spectral density
