# Supplementary material for: Role of meteorological conditions in reported chickenpox cases in Wuhan and Hong Kong, China
Source: BMC Infect Dis. 2017 Aug 3;17:538. doi: 10.1186/s12879-017-2640-1 (PMC5541728; doi:10.1186/s12879-017-2640-1)

# **Additional file 1**

**Plot of *Q*_1_ and *Q*_2_ against mean wind velocity. The black filled circles show *Q*_1_ for Japan’s 47 prefectures, the black open circles *Q*_2_ for Japan’s 47 prefectures, the red filled circles *Q*_1_ for Wuhan, and red open circles *Q*_2_ for Wuhan.**


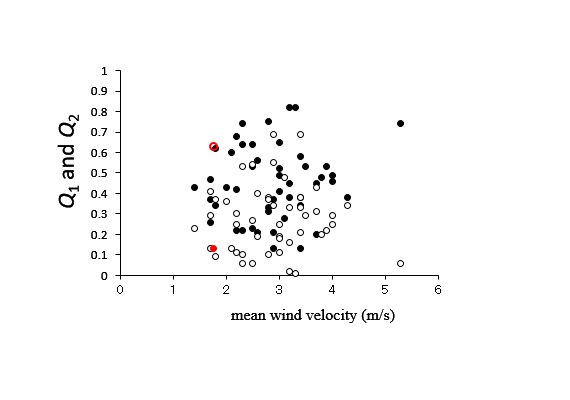

Supplement: Supplementary file 1 — Plot of Q 1 and Q 2 against mean wind velocity. The black filled circles show Q 1 for Japan’s 47 prefectures, the black open circles Q 2 for Japan’s 47 prefectures, the red filled circles Q 1 for Wuhan, and red open circles Q 2 for Wuhan. (DOCX 23 kb) [file 12879_2017_2640_MOESM1_ESM.docx]
