# Supplementary material for: Role of meteorological conditions in reported chickenpox cases in Wuhan and Hong Kong, China
Source: BMC Infect Dis. 2017 Aug 3;17:538. doi: 10.1186/s12879-017-2640-1 (PMC5541728; doi:10.1186/s12879-017-2640-1)

# **Additional file 2**

**Plot for *Q*_1_ and *Q*_2_ of the monthly data for Wuhan compared with the results obtained with the corresponding weekly data shown in Figure 4. The red filled circles indicate *Q*_1_ and *Q*_2_ for Wuhan’s monthly data. To convert the original weekly data into monthly data,** t**he weekly data extending across two months from the end of the first month to the beginning of the next month were decomposed into daily data by calculating the mean daily values of the weekly data. Then, we obtained monthly data by adding the daily data thus obtained and the remaining weekly data.**


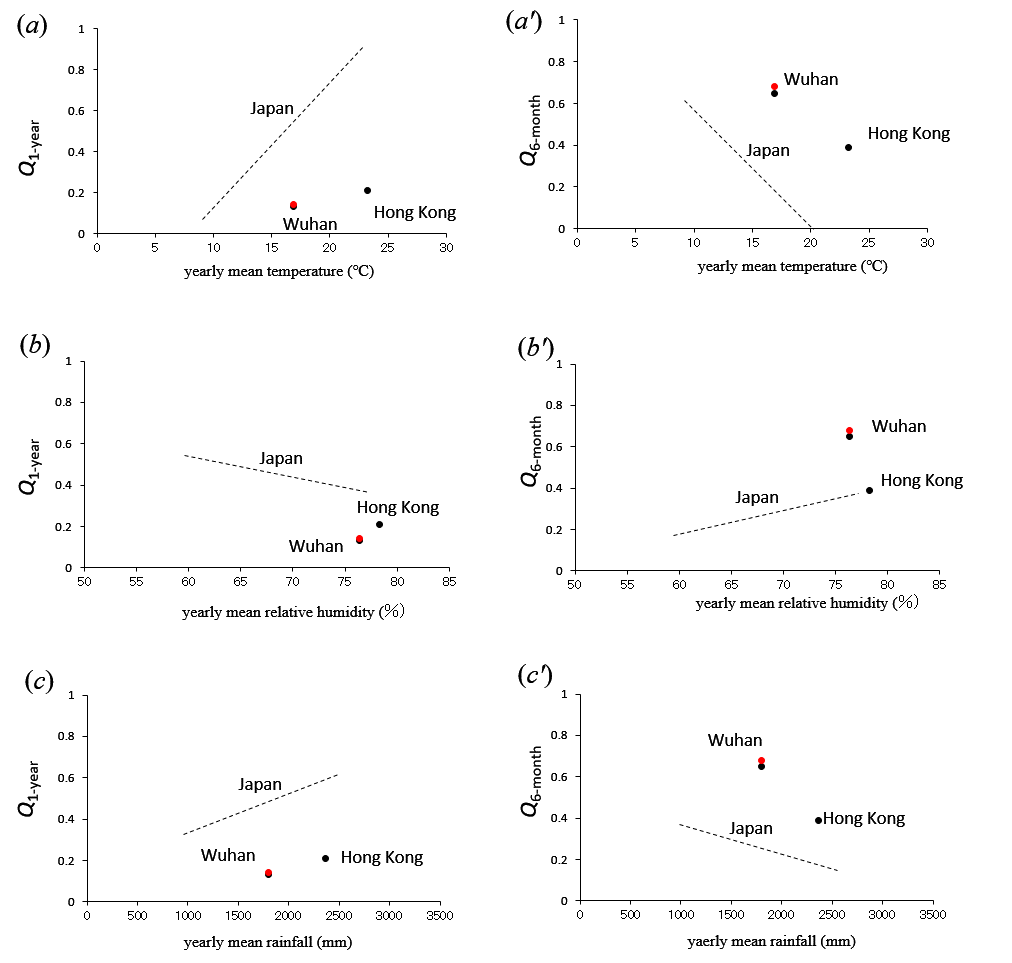

Supplement: Supplementary file 2 — Plot for Q 1 and Q 2 of the monthly data for Wuhan compared with the results obtained with the corresponding weekly data shown in Fig. 4. The red filled circles indicate Q 1 and Q 2 for Wuhan’s monthly data. To convert the original weekly data into monthly data, the weekly data extending across two months from the end of the first month to the beginning of the next month were decomposed into daily data by calculating the mean daily values of the weekly data. Then, we obtained monthly data by adding the daily data thus obtained and the remaining weekly data. (DOCX 46 kb) [file 12879_2017_2640_MOESM2_ESM.docx]
